# Texture Analysis of Abnormal Cell Images for Predicting the Continuum of Colorectal Cancer

**DOI:** 10.1155/2017/8428102

**Published:** 2017-01-17

**Authors:** Ahmad Chaddad, Camel Tanougast

**Affiliations:** Laboratory of Conception, Optimization and Modelling of Systems, University of Lorraine, 7 rue Marconi, Metz, 57070 Lorraine, France

## Abstract

Abnormal cell (ABC) is a markedly heterogeneous tissue area and can be categorized into three main types: benign hyperplasia (BH), carcinoma (Ca), and intraepithelial neoplasia (IN) or precursor cancerous lesion. In this study, the goal is to determine and characterize the continuum of colorectal cancer by using a 3D-texture approach. ABC was segmented in preprocessing step using an active contour segmentation technique. Cell types were analyzed based on textural features extracted from the gray level cooccurrence matrices (GLCMs). Significant texture features were selected using an analysis of variance (ANOVA) of ABC with a *p* value cutoff of *p* < 0.01. Features selected were reduced with a principal component analysis (PCA), which accounted for 97% of the cumulative variance from significant features. The simulation results identified 158 significant features based on ANOVA from a total of 624 texture features extracted from GLCMs. Performance metrics of ABC discrimination based on significant texture features showed 92.59% classification accuracy, 100% sensitivity, and 94.44% specificity. These findings suggest that texture features extracted from GLCMs are sensitive enough to discriminate between the ABC types and offer the opportunity to predict cell characteristics of colorectal cancer.

## 1. Introduction

Colorectal cancer (CRC) represents one of the most frequent cancers affecting people [1]. It is characterized by abnormal and uncontrolled cellular proliferation [2]. Surgical resection of the primary tumor with curative intent is possible in only 70% of patients. Unfortunately, up to 30% of CRC patients who undergo surgical resection of the primary tumor experience a subsequent relapse within 3 years and with a median time to death of 12 months [3, 4]. Colorectal cells are transformed by CRC into anomalous and heterogeneous shapes [5, 6]. In this context, heterogeneity is a pronounced feature of colorectal cancer that manifests as areas of high cell density. Attempts to quantify heterogeneity have been made using multiple feature functions such as Haralick features [6]. Another instance has used the link between the texture of hepatic tissue and its entropy and uniformity to predict survival using computer tomography images [7]. However, limited studies have used texture features to assess the continuum of CRC from benign to malignant cells.

Additionally, classical optical microscopy systems can detect ABC by applying advanced image processing techniques [8]. Early detection of ABC by shape or heterogeneity is of high interest in order to diagnose and start therapy early [6]. Hence, automating the process allows a faster and more precise reading of microscopic biopsies and may even allow classification of samples as BH, IN, or Ca [6, 9, 10]. In this context, numerous studies have considered developing automated reading procedures of such biopsies [5, 11–14]. The biopsies examined by these procedures can be prepared and preprocessed for automated reading using the optical microscopy system. Then, ABCs can be analyzed from their surrounding media using segmentation techniques [15]. In this context, the appropriate segmentation technique must be carefully established in order to process multispectral bioimages from microscopic system that provides high resolution gray scale images. Moreover, identification of ABC within an image should take into consideration some characteristic features that are representative of each ABC type [6]. Texture feature extraction from ABC can be a promising technique to characterize each ABC type. Then, discrimination between ABC types can be done by applying one of the classifier approaches such as the decision tree approach [16]. The analysis of the textures and structures of each ABC type permits a more accurate diagnosis of the malignant cells as they are structured in various patterns and textures.

In this work, we propose to analyze each of ABC type by extracting texture features from GLCM. Texture refers to the variability in tone within a region, or the spatial relationships among the gray levels of neighboring pixels. Three-dimensional (3D) texture analyses offer more information by using two phases and multioffset pixels to detect the variability of pixel pairs in 3D space [16, 17]. The statistical approach of image analysis based on the matrix of cooccurrence is commonly applied to optical and medical images to evaluate morphology [5, 11, 18, 19]. The texture features thus extracted from GLCM describe the texture and local variation in an image. For classification, we selected twelve principal features in order to identify ABC types, while discarding those that are either redundant or confusing, thereby improving the performance of the proposed feature based detection technique.

In summary, the purpose of this study is the derivation of quantitative texture multispectral image features from optical microscopy images that classify the continuum of CRC lesions. The novelty of this study was the first training on an automated continuum prediction of CRC. This will be foundation of radiomic maps that associate these texture features with various ABC types.

The remainder of this paper is organized as follows.  Section 2 describes the texture feature extraction from 3D GLCM in detail with performance metrics. Sections 3 and 4 demonstrate experimental results and discussions. Finally, Section 5 concludes the paper.

## 2. Materials and Methods

**Figure 1:**
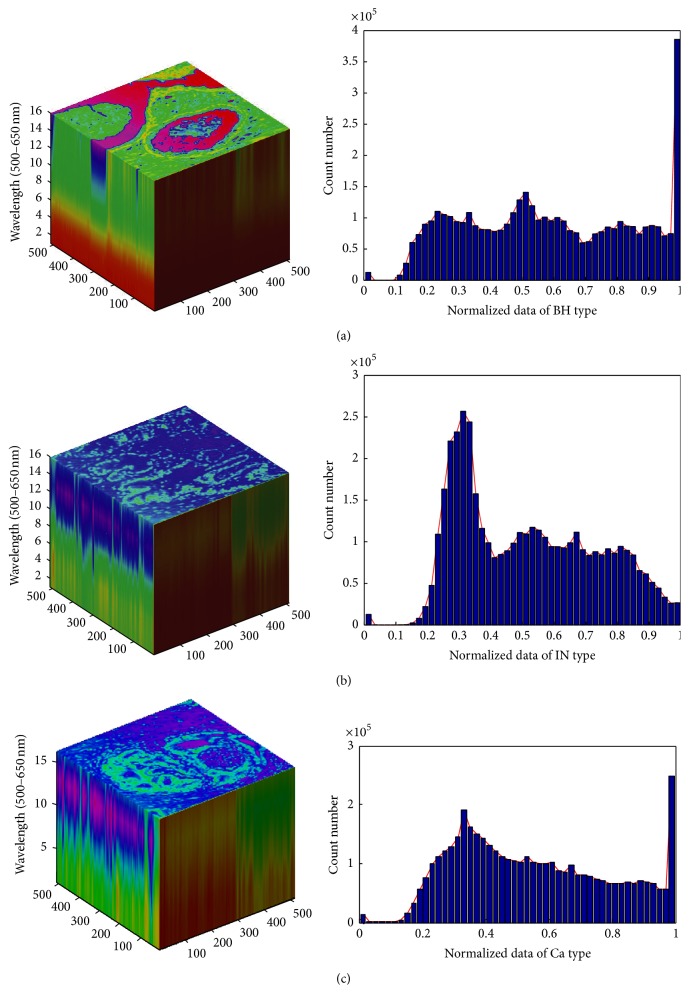
Multispectral images of three types from abnormal cells showed differences in shape and histograms. These images come from colorectal biopsies viewed under optical microscopy systems (×40 objective magnification). (a) Benign hyperplasia, (b) intraepithelial neoplasia, and (c) carcinoma.

We specifically analyzed 3D multispectral digital whole slide images (WSI) from 27 colorectal cancer patients. An example of spatial heterogeneity for each multispectral ABC type is seen in its histogram distribution. Clearly, there are certain characteristics and features from preliminary analysis that differentiate BH, IN, and Ca (Figure 1).

### 2.1. Sample Preparation and Data Acquisition

Whole tissue samples were taken from colonic glands with thickness value of 5 *μ*m which is stained using Haematoxylin and Eosin (H & E) stains. Images were captured by a charge coupled device (CCD) camera integrated with a liquid crystal tunable filter (LCTF) in the optical microscopy system [20]. LCTF provides multispectral images of the tissue samples by changing the wavelength operation [21]. LCTF has a bandwidth of 5 nm, and its wavelength is controllable through the visible spectrum range of 400–720 nm. Multispectral images are produced through repeated image capture in various wavelengths subbands. Moreover, the impact of multispectral imaging has been shown that the classifier accuracy increases with the number of spectral bands [22]. Note that each image band is 8 bits coded and hence has 255 possible light intensity levels.

In this study, LCTF offered 16 multispectral bands using a wavelength range of 500–650 nm. Thus, from each original image, we obtained 16 images representing the wavelength range and a volume of multispectral data (Figure 1). Hence, texture extraction from each band of multispectral data enhances the lesion characterizing each abnormal cell type. Note that a colorectal pathologist views images at lower power to identify the abnormal cells which is represented by a low magnification (×40) of image samples.

### 2.2. Patients

**Figure 2:**
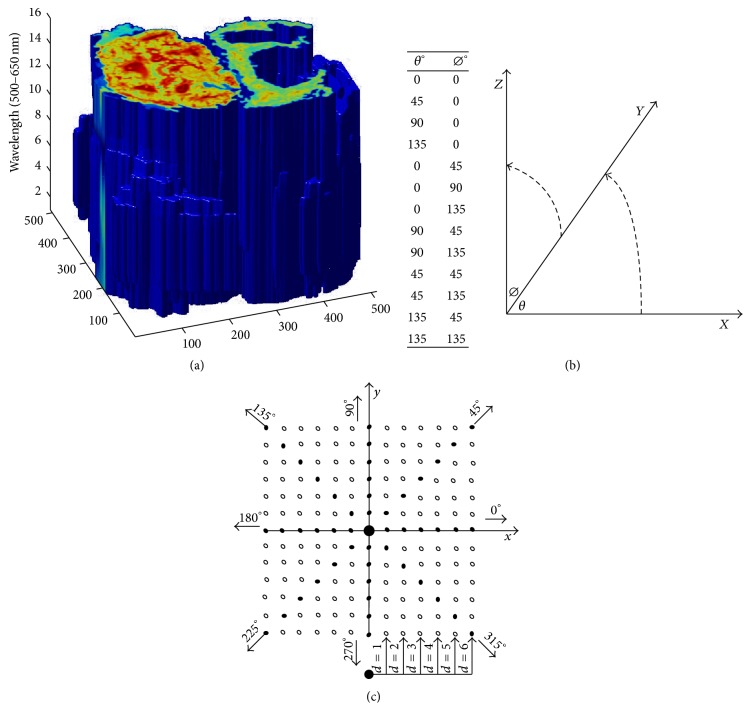
Volume data of multispectral bioimages are shown above. (a) Segmented multispectral gray scale images of carcinoma are analyzed pixel by pixel. In (b), 13 directions controlled by two angles (*θ*°, *Ø*°), where *x* and *y* are the image coordinates and *z* is the wavelength range of 500–650 nm. (c) A computation map of GLCM shows the distance with direction in 2D.

After excluding samples with incomplete data, a set of 27 CRC patients were gathered for a preliminary study. We selected nine volumes of data from each ABC type, where a volume of data was structured in 16 multispectral images (Figure 1). Thus, images were filtered by an average filter (spatial filter) before further segmentation processing to minimize the effects of noise in images and other external factors. All the images were reconstructed to a 512 × 512 matrix where the volume size of 512 × 512 × 16 was taken into consideration in texture feature extraction from GLCM of ABC (Figure 2(a)).

### 2.3. Segmentation of Abnormal Cell

**Figure 3:**
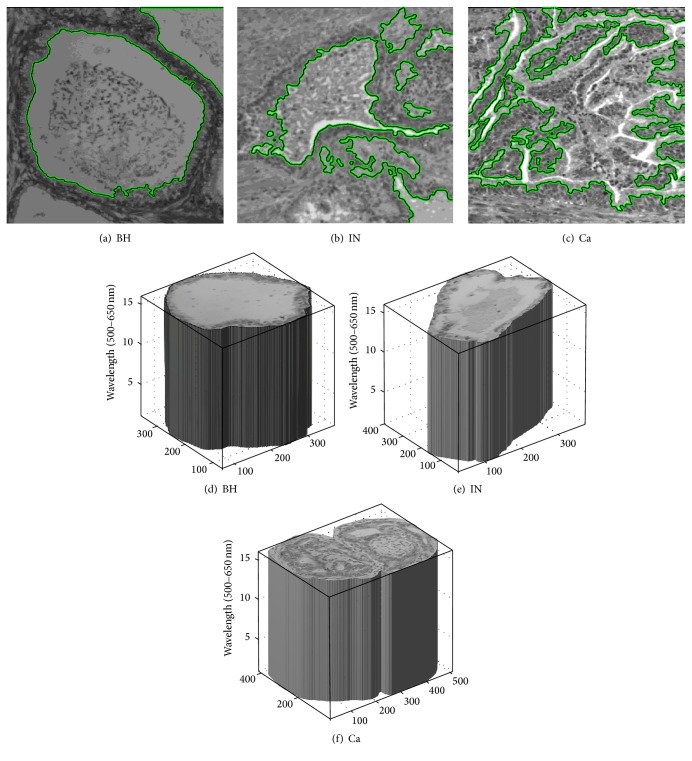
The effect of the snake segmentation technique for 2D multispectral bioimages was applied to the three ABC types (a, b, and c). Schema of 3D multispectral bioimages segmented after area determination from colorectal pathologist (d, e, and f).

We employed active contour segmentation to accurately segment anomalous shape of cells. This technique is based on a dynamic curve that moves toward and detects the contour of the object by a number of iterative processes [23, 24]. This approach was successfully implemented to detect ABC types from similar kinds of multispectral bioimages. The computation time was improved by limiting the number of iterations, which was set automatically based on empirical calculations [6]. Computation time was further strengthened by resizing the images. For instance, an image of size 512 × 512 pixels was decreased to 64 × 64 pixels and active contour was applied to detect cells within the image. Active contour images were then resized to 512 × 512 pixels and placed on the original image (Figure 3). In fact, this technique resized the active contour and not the original image in order to enhance the computation time. Cell images in 16 multispectral images were then assessed by a board certified colorectal pathologist. A volume of a cell segmented in 16 multispectral images (2D images) was created to represent the variance details in the multispectral band (Figure 2(a)). To assess the active contour segmentation, ground truth of cell and cells segmented based on active contour were considered. In this context, evaluation of WSI segmentation considered two similarity metrics, namely, Jaccard similarity coefficient (JSC) and Dice similarity coefficient (DSC). Additionally, false positive rate (FPR) and false negative rate (FNR) were also computed. JSC and DSC measure the degree of the correspondence between ground truth cell images and segmented images.

JSC can be formulated according to the following:

(1) $$\begin{array}{c} \text{JSC}\left(\;A,B\;\right)=\frac{\left(A\cap B\right)}{\left(A\cup B\right)}, \end{array}$$

where *A* and *B* are the area of ground truth of cell and segmented cell, respectively.

JSC was employed to calculate the overall level of similarity between segmented cell and ground truth cell. DSC was also employed and can be expressed according to the following:

(2) $$\begin{array}{c} \text{DSC}\left(\;A,B\;\right)=\frac{2\left(A\cap B\right)}{\left|A\right|\cup \left|B\right|}. \end{array}$$

Additionally, we employed FPR and FNR which were used to quantify over- and undersegmentation. Both FPR and FNR are calculated according to the following:

(3) FPRA,B=A/BA∪B,FNRA,B=B/AA∪B.

Direct relation between JSC, FPR, and FNR is defined according to the following expression:

(4) $$\begin{array}{c} \text{JSC}\left(\;A,B\;\right)=1-\text{FPR}-\text{FNR}. \end{array}$$

The performance metrics of active contour technique were reported (Table 1). This volume of WSI was quantified by the texture feature extracted from GLCMs of each abnormal cell type.

### 2.4. GLCM Based Feature

One of the best techniques used to evaluate the relationships between image pixels is the texture feature extraction from GLCM. This technique was proposed by Haralick et al. in 1973 [19]. It is one of the most popular second-order statistical features which is based on GLCM computation and its texture features. Then, second-order statistics estimate properties of two or more pixel values occurring at specific locations relative to each other. For these reasons, we proposed to use GLCM based feature technique in this work.

#### 2.4.1. Gray Level Cooccurrence Matrix (2D and 3D)

GLCM represents the probabilities *P*_*d*,*θ*_(*i*, *j*) of transition from a pixel with intensity “*i*” to a pixel of intensity “*j*” separated by a translation vector defined by direction “*θ*” and an offset “*d*” (offset known as distance) [11, 16–19]. Given a two-dimensional (2D) image *I* of size *N* × *N*, the cooccurrence matrix *P*_*d*,*θ*_(*i*, *j*) can be defined as follows:

(5) Pd,θi,j=∑x=1N∑y=1N1,if  Ix,y=i,  Ix+dx,y+dy=j,0,otherwise,

where *dx* and *dy* specify the distance between the pixel of interest and its neighbor, along the *x*-axis and the *y*-axis of an image, respectively. GLCM is a square matrix of size *Ng*, where *Ng* is the number of gray levels in the image.

For 2D images, typical values used for “*d*” equal {1,2, 3,4} and those for “*θ*” equal {0°, 45°, 90°, 135°}. The GLCMs corresponding to the additional directions {180°, 225°, 270°, 315°} do not add to the specification of the texture already caught by the 16 GLCMs associated with combinations of the aforementioned four offsets and four directions. This is because there is symmetry in GLCMs described by (*P*(*d*, 0°) = *P*^*T*^(*d*, 180°); *P*(*d*, 45°) = *P*^*T*^(*d*, 225°); *P*(*d*, 90°) = *P*^*T*^(*d*, 270°); and *P*(*d*, 135°) = *P*^*T*^(*d*, 315°)) (note that superscript “*T*” denotes the transpose operation).

GLCM computations can be also applied to 3D images. In this case, the GLCM *P*(*i*, *j*) counts the number of pixel pairs that have intensities “*i*” and “*j*” for the spatial relationship specified by a translation vector (*dx*, *dy*, *dz*), where *dx*, *dy*, and *dz* represent the number of pixel offsets along the *x*-axis, *y*-axis, and *z*-axis of the 3D image. For volumetric data, two angles (*θ*, *Ø*) lead to 13 directions (Figure 2). Each segmented cell was histogram equalized to 32 levels, and then we employed the GLCM computation.

The foremost advantage of GLCMs applied to volumetric data is the ability to capture intensity relationships between the pixels in a 3D volume. Further, the number of GLCMs resulting from 3D operations is typically smaller than that corresponding to numerous 2D slices. For example, in a data cube with 10 separate 2D slices, there are a total of 80 GLCMs (8 GLCMs analogous to 2 offsets and 4 directions per slice). On the other hand, in a 3D operation, the total number of GLCMs is 26 (13 directions and 2 offsets). Supported by the benefit of GLCMs applied on volumetric data, we computed GLCMs of multispectral ABC and quantified these cooccurrence matrices by Haralick features.

#### 2.4.2. Texture Quantification

Haralick proposed 14 texture features to be extracted from GLCMs, and the value of each extracted feature indicates the preliminary indicators of ABC in the texture image. Among the 14 texture features, we employed the 12 principal textural features: energy (*f*_1_), entropy (*f*_2_), correlation (*f*_3_), contrast (*f*_4_), homogeneity (*f*_5_), variance (*f*_6_), sum-mean (*f*_7_), inertia (*f*_8_), cluster shade (*f*_9_), cluster tendency (*f*_10_), maximum probability (*f*_11_), and inverse difference moment (*f*_12_). These features are defined by their functions as follows

(6) $$\begin{array}{c} f_{1}=\overset{Ng}{\underset{i=1}{\sum }}\overset{Ng}{\underset{j=1}{\sum }}\left(\;ij\;\right)P_{d,\theta }{\left(\;i,j\;\right)}^{2} \end{array}$$

shows the scale of texture homogeneity. It is high when the GLCMs consist of few pixels of high amplitude and low when all the values of GLCMs are almost similar.

(7) $$\begin{array}{c} f_{2}=-\overset{Ng}{\underset{i=1}{\sum }}\overset{Ng}{\underset{j=1}{\sum }}P_{d,\theta }\left(\;i,j\;\right)\log\left(\;P_{d,\theta }\left(\;i,j\;\right)\;\right) \end{array}$$

measures the disorder or complexity of an image. The highest value of entropy is found when the values of *P*(*i*, *j*) are allocated quite uniformly throughout the matrix.

(8) $$\begin{array}{c} f_{3}=\frac{\sum_{i=1}^{Ng}\sum_{j=1}^{Ng}\left(ij\right)P_{d,\theta }\left(i,j\right)-\mu_{x}\mu_{y}}{\sigma_{x}\cdot \sigma_{y}} \end{array}$$

measures the linear dependence of gray level values in the GLCM or describes the correlations between the rows and columns of GLCM.

(9) $$\begin{array}{c} f_{4}=\overset{Ng-1}{\underset{n=0}{\sum }}n^{2}\left\{\;\overset{Ng}{\underset{i=1}{\sum }}\overset{Ng}{\underset{j=1}{\sum }}P_{d,\theta }\left(\;i,j\;\right)\;|\;\left|\;i-j\;\right|=n\;\right\} \end{array}$$

measures intensity contrast or the local variations present in an image to show the texture fineness.

(10) $$\begin{array}{c} f_{5}=\overset{Ng}{\underset{i=1}{\sum }}\overset{Ng}{\underset{j=1}{\sum }}\left(\;\frac{P_{d,\theta }\left(i,j\right)}{{\left(1+\left|i-j\right|\right)}^{2}}\;\right) \end{array}$$

returns a value that measures the closeness of the elements distribution in GLCM to the GLCM diagonal.

(11) $$\begin{array}{c} f_{6}=\overset{Ng}{\underset{i=1}{\sum }}\overset{Ng}{\underset{j=1}{\sum }}{\left(\;i-\mu_{x}\;\right)}^{2}P_{d,\theta }\left(\;i,j\;\right)+{\left(\;j-\mu_{y}\;\right)}^{2}P_{d,\theta }\left(\;i,j\;\right) \end{array}$$

is expected to be large if the gray levels of the image are spread out greatly.

(12) $$\begin{array}{c} f_{7}=\overset{2Ng}{\underset{n=2}{\sum }}n\left(\;\overset{Ng}{\underset{i=1}{\sum }}\overset{Ng}{\underset{j=1}{\sum }}P_{d,\theta }\left(\;i,j\;\right)\;\right) \end{array}$$

measures the average of the gray levels. It can be high value if the sum of the gray level of the image is high.

(13) $$\begin{array}{c} f_{8}=\overset{Ng}{\underset{i=1}{\sum }}\overset{Ng}{\underset{j=1}{\sum }}{\left|\;i-j\;\right|}^{2}\cdot P_{d,\theta }\left(\;i,j\;\right) \end{array}$$

measures the inhomogeneous in image.

(14) $$\begin{array}{c} f_{9}=\overset{Ng}{\underset{i=1}{\sum }}\overset{Ng}{\underset{j=1}{\sum }}{\left(\;i+j-\mu_{x}-\mu_{y}\;\right)}^{3}P_{d,\theta }\left(\;i,j\;\right) \end{array}$$

measures the skewness (asymmetric) of the GLCM and is considered to gauge the perceptual concepts of uniformity. When the cluster shade is high, the image is asymmetric.

(15) $$\begin{array}{c} f_{10}=\overset{Ng}{\underset{i=1}{\sum }}\overset{Ng}{\underset{j=1}{\sum }}{\left(\;i+j-\mu_{x}-\mu_{y}\;\right)}^{2}P_{d,\theta }\left(\;i,j\;\right) \end{array}$$

measures the grouping of pixels that have similar gray level values.

(16) $$\begin{array}{c} f_{11}=\underset{i,j}{\max}\left(\;P_{d,\theta }\left(\;i,j\;\right)\;\right) \end{array}$$

measures the dominant pair pixels in the GLCM. It can be high if the dominant pair pixel is high.

(17) $$\begin{array}{c} f_{12}=\overset{Ng}{\underset{i=1}{\sum }}\overset{Ng}{\underset{j=1}{\sum }}\frac{P_{d,\theta }\left(i,j\right)}{1+{\left(i-j\right)}^{2}} \end{array}$$

measures the smoothness of the image. It can be high if the gray levels of the pixel pair are similar.

For the ABC detection problem, the aforementioned textural features are extracted from the 3D GLCMs conforming to the 13 directions and 4 types of offset. Therefore, the length of the resulting feature vector is 12 (functions) × 13 (directions) × 1 (distance or offset) = 156 features. To analyze the effect of texture feature based on GLCMs, we organized texture features into 5 groups (G_1_, G_2_, G_3_, G_4_, and G_5_) reported in Table 2.

Moreover, we calculated the average of texture feature based on 3D GLCM within 13 directions and 4 offsets to evaluate the value of each one from ABC (Table 3). Additionally, we employed feature selection techniques on each texture feature group to demonstrate the effectiveness of texture analysis in a definite direction and offset, and the performance metrics were reported (Table 4).

### 2.5. Statistical Analysis

Textures quantified by twelve functions (based on those suggested by Haralick) can be found among the BH, IN, and Ca cell samples. *Z*-score normalization was employed on each of the feature vectors, which converted the features to zero mean and unit variance [25]. The mean and standard deviation (*σ*) of the feature vector are calculated as follows:

(18) $$\begin{array}{c} r_{n}=\frac{r-\text{mean}}{\sigma }, \end{array}$$

where *r* is the original value, *r*_*n*_ is the new value, and the mean and *σ* are the mean and standard deviation of the original data, respectively.

ANOVA was used to assess the statistical significance between texture features and ABC types [26]. This test was used to identify the significant texture feature where a *p* value < 0.01 was deemed significant. An aggregate of 158 significant features were selected, which was further reduced using PCA. Five principal components (PCs) representing 97% of the variance among the 158 selected features were used in a decision tree classifier (Tables 3 and 4).

### 2.6. Classifier Setting and Performance Metrics

Classification of the ABC types based on texture features was performed using the significant features as input variables in a decision tree (DT) classifier [27]. The most important aspect of a decision tree induction strategy is the split criteria; it is a method of selecting an attribute that determines the distribution of training objects into subsets upon which subtrees are consequently built. In this study, a goodness criterion based on Gini index was used to determine how well various feature test conditions performed [28]. The reason to use the DT classifier is to find automatically the dominant features and provide the classifier metrics; however, we considered the naïve Bayes and nearest neighbors to evaluate the classifier performance metrics using the known class labels from ABC types. Due to limited data (27 patients), the classifier was validated using leave-one-out cross-validation [29]. We considered the following performance metrics of classification: accuracy, sensitivity, specificity, *F*-score, and area under the curve (AUC), which were performed to test the reliability of the texture feature classifier. We used multiple metrics for better assessing the feasibility of abnormal cell type discrimination using texture feature based 3D GLCMs. Note that true positive (TP) and true negative (TN) are the number of positive and negative samples correctly classified; false positive (FP) and false negative (FN) are the number of positive and negative samples incorrectly classified [30]. Then, TP + FN is the total number of test samples of the considered class. For example, TP of BH cell type represents the BH samples correctly classified and FN represents the BH cell type incorrectly classified. Also, FP of BH cell type represents the IN and Ca samples classified as BH cell type, while TN of BH cell type represents the number of correctly classified IN and Ca samples and number of IN samples classified as Ca and the number of Ca samples classified as IN cell type.

Accuracy represents the correctly classified samples and can be expressed by the following:

(19) $$\begin{array}{c} \text{Accuracy}=\frac{\text{TP}+\text{TN}}{\text{TP}+\text{FP}+\text{TN}+\text{FN}}. \end{array}$$

Sensitivity is a measure of the capability of a classifier to recognize the positive class patterns. It can be expressed according to the following:

(20) $$\begin{array}{c} \text{Sensitivity}=\frac{\text{TP}}{\text{TP}+\text{FN}}. \end{array}$$

Specificity is a measure of the capability of a classifier to recognize the negative class patterns. It can be expressed by the following:

(21) $$\begin{array}{c} \text{Specificity}=\frac{\text{TN}}{\text{TN}+\text{FP}}. \end{array}$$

*F*-score is a weighted average of precision and recall and can be calculated using the following:

(22) $$\begin{array}{c} F\text{-score}=\frac{2\times \text{TP}}{\left(2\times \text{TP}+\text{FP}+\text{FN}\right)}. \end{array}$$

## 3. Experimental Results

ABC digital images were segmented using the active contour segmentation technique. Figure 3 shows ABC types segmented using several steps. The process of cell detection from multispectral images may appear to be a difficult task as bioimages contained some areas that have a similar range of gray shades and irregular shapes. Morphology operators were necessary to select the required cells from images by a board certified colorectal pathologist because there were multiple cell types within images.

Snake (active contour) techniques showed that ABC types were correctly detected and located (Figure 3). JSC shows a similarity range of 75.92–81.56% with the best performance achieved with Ca cell type. Meanwhile, DSC shows a similarity range of 86.31–88.21% with the best performance achieved with Ca cell type. Moreover, FPR shows a range of 05.03–07.61% with the best performance achieved with IN cell type, while we observed that FNR provided a range value of 16.11–20.26% with the best performance achieved with Ca cell type (Table 1). These metrics confirmed the feasibility of active contour segmentation method to determine the abnormal cell types and specifically the Ca cell type (Table 1).

**Figure 4:**
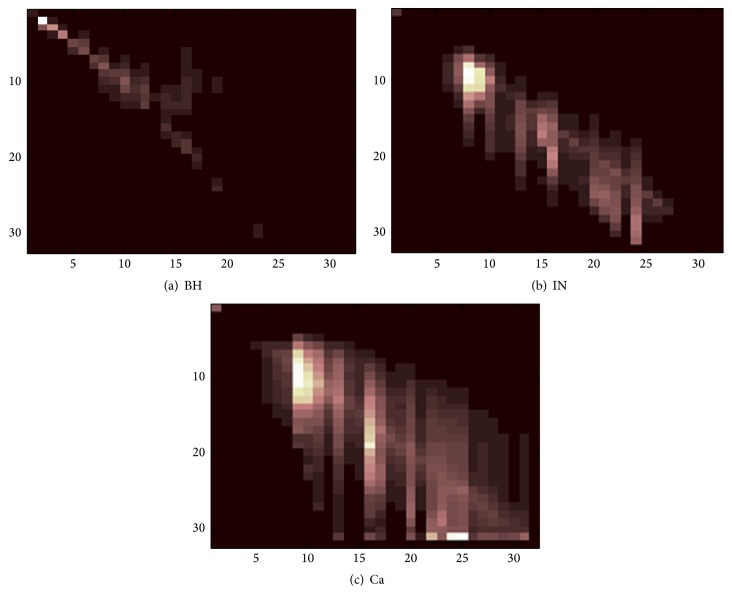
GLCM (32 gray levels) of multispectral bioimages is shown for all three ABC types. GLCM is in one direction (*θ* = 0°, *Ø* = 0°) and distance is constant (*d* = 1, one pixel offset).

Figure 4 shows an example case of GLCMs for corresponding ABC types in Figure 3. GLCM images showed the most pronounced texture associated with Ca cells among the three ABC types. These texture values represent a high number of pixel pairs in the original image of Ca cells, followed by IN and BH cells, respectively. Additionally, BH images had a homogenous texture that was more homogenous than IN and Ca; its corresponding GLCM showed that most BH textures were depicted in the diagonal of the GLCM image. Notably, when the texture GLCM image has more fitted data around the diagonal, the original image is less homogenous. The average of texture functions based on whole offsets and directions showed the differences between the ABC groups, which were demonstrated in each of the 12 texture features extracted from GLCMs (as shown in Table 3). For instance, energy (*f*_1_) exhibited a higher value (0.014) in BH followed by IN and Ca cells, respectively. This is demonstrated by the high homogeneity of BH cells. Entropy (*f*_2_) showed close values between the ABC groups with a maximum value of 2.53 for Ca cells. This is reflected by the disorder of texture in Ca cells, but the close values of ABC types can be a weak classifier feature. Thus, correlation (*f*_3_) showed a maximum value of 0.016 for IN cells; this is reflected by the higher dependence of gray level in IN GLCM images. Compared to all texture features of ABC types, the maximum value of features (*f*_1_, *f*_4_, *f*_5_, *f*_8_, *f*_10_, and *f*_11_), (*f*_3_ and *f*_12_), and (*f*_2_, *f*_6_, *f*_7_, and *f*_9_) was found for BH, IN, and Ca type, respectively. With the exception of feature (*f*_6_), significant differences between the ABC types were noted in all the texture features examined (*p* value < 0.01).

An ANOVA showed that there were 35 significant classifier features among the 156 texture features for G_1_, G_2_, and G_3_, while G_4_ provided 53 significant features for discrimination between ABCs. The total number of significant features was 158 (G_5_) by simple concatenation of group features (Table 4).

Performance metrics range of ABC discrimination based on specific offset and direction showed accuracy, sensitivity, and specificity ranges of 55.55–77.77%, 55.55–88.88%, and 66.66–94.44%, respectively.

Maximum values of the performance metrics range were achieved by using group G_3_ which represented a four-pixel offset and 13 directions of GLCMs (Table 4). Moreover, classifier accuracy for each ABC feature exhibited a range of 55.55–88.88%, 44.44–88.88%, and 55.55–66.66% for BH, IN, and Ca, respectively (Table 5).

Furthermore, five PCs features showed the highest value of 92.59% accuracy, 100% sensitivity, and 94.44% specificity (the last row in Table 4).

The highest classifier accuracy obtained for IN was 88.88% using G_1_ and G_2_ features (Table 5). However, BH and Ca features showed the highest values of 100% using five PCs features (Table 5).

**Figure 5:**
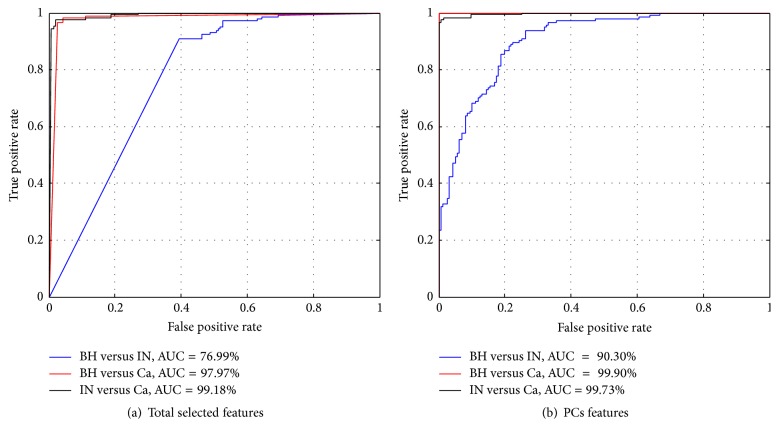
Receiver operating characteristic (ROC) curves were determined for each discrimination between ABC (BH versus IN, BH versus Ca, and IN versus Ca) problems based on real 3D data. The area under the curve (AUC) values achieved are shown for each discrimination between ABCs in two cases: (a) total selected feature used is 158 and (b) PCs features used are 5.

The analysis of the ROC curve obtained for each ABC discrimination showed ranges of AUC values of 76.99–90.30% for BH versus IN, 97.97–99.90% for BH versus Ca, and 99.18–99.73% for IN versus Ca, respectively. However, the best AUC values for ABC discrimination were achieved using 5 PCs features (Figure 5).

Moreover, using five PCs features, comparative study of the confusion matrix for abnormal cell type discrimination based on decision tree (DT), naïve Bayes (NB), and nearest neighbors (NN) classifier [26–28] showed that the nine BH and nine Ca samples are correctly classified based on DT and NB classifier, respectively. However, eight IN samples are correctly classified based on NB classifier technique (Table 6).  *F*-score showed the highest BH, IN, and Ca metric with 94.73, 94.11, and 100%, respectively, using NB classifier. This demonstrates that the best classifier technique for discriminating BH from IN and Ca is NB (Table 7).

## 4. Discussion

In this study, we have shown the role of texture feature extraction from GLCMs to discriminate BH, IN, and Ca. We demonstrated the use of quantitative image texture features and reported significant features with performance metrics for ABC discrimination. Additionally, we showed the power of texture quantification from GLCMs using 12 functions to indirectly associate image features with ABC types. Texture features extracted from GLCMs using four pixels offsets and 13 directions (G_3_) showed a higher accuracy to discriminate between types of ABC than other features groups. This proves that the GLCMs of fourth pixel neighbors in 13 directions can offer the best automated ABC classification (Table 4). Similarly, classifier features of BH showed effectiveness to identify BH cells by texture features extracted from GLCMs using 4 pixels offsets and 13 directions (G_3_ in Table 5). Abnormal IN cell presented the best classification using G_1_ and G_2_, and Ca shows the best classification using G_2_, G_3_, and G_5_. However, without using PCs features, a lack of predictive accuracy of Ca demonstrated that its texture may resemble the texture of IN, which represents the complexity of malignant diagnostic (Table 5).

**Figure 6:**
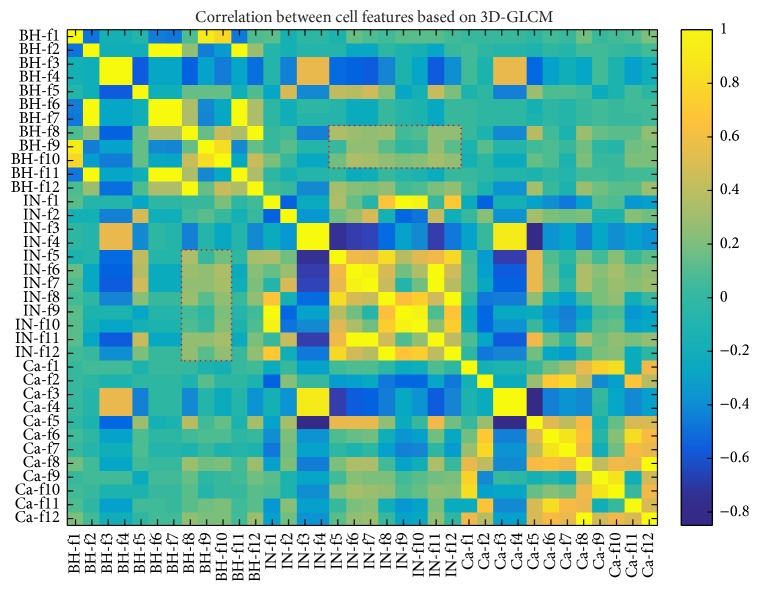
Heat map with correlation coefficients between ABC features: BH, IN, and Ca are the begin hyperplasia, intraepithelial neoplasia, and carcinoma, respectively. Red rectangular is the correlation effect of ABC feature which decreased the accuracy value of ABC discrimination.

According to the experiments in which different groups of texture feature were applied to the ABC discrimination process, the results showed the efficiency of PC features derived from significant texture features extracted using GLCMs for histopathology colorectal cancer image analysis (last row of Tables 4 and 5). This potential of PC features demonstrated the highest AUC values for discrimination of BH versus IN, BH versus Ca, and IN versus Ca (Figure 5).  Figure 6 showed a heat map correlation between the ABC features where the highest correlation represents the resemblance between the texture features. We observed that some BH and IN features (red rectangular shape) have a high correlation value. This represented the lack of performance metrics.

This study demonstrates that texture feature extraction can be a map for ABC identification by using the techniques in image processing such as significant feature and feature selection. Previously, differentiation of human colon cancer cells was demonstrated using gene expression of B-tubulin isotypes [31]. More recently, multilabel classification of colon cancer using histopathological images was performed using several types of features. It was concluded that combined features can offer good performance for multilabel colon cancer prediction, with a precision of 73.7% [32]. Moreover, another study has proven that colon cancer prognosis can be identified by using distinct molecular subtypes and serrated precursor lesions [33]. Thus, the effort to analyze the continuum of colorectal cancer is still incomplete.

To date, few studies have directly addressed the discrimination between types of ABC for the diagnosis of colon cancer. Most have focused on their heterogeneity; several studies have suggested that increasing heterogeneity is associated with malignancy [34]. Additionally, it has been proposed that greater biologic heterogeneity may be associated with oxidative stress and genomic instability [35]. Also, a study based on hepatic texture in patients with CRC found a more heterogeneous liver texture at coarse scale (textures extracted based on Laplacian of Gaussian filter) is related to the presence of occult malignancy [36]. Moreover, in this work, it was proven that higher value of entropy function is associated with carcinoma which represents a higher heterogeneity between ABC types.

This study offers a simple approach based on texture feature analysis to evaluate the continuum of colorectal cancer from benign to malignant by using three abnormal cells. These three cell types represent the transformation from benign to malignant cancer. In this context, the results showed that radiomic texture feature is significant and provide good classifier metrics and also highlight the potential of radiomic texture feature extraction for enhanced prediction of ABC from colorectal tissues. This should trigger further research of image-based quantitative texture features in colorectal cancer. Given the reality that colorectal cancer is highly heterogeneous between patients, texture feature analysis is a more desirable approach to provide clear categorization of ABC type than the established methods.

Our study had limitations; the most important of these was the limited subjects (*n* = 27). Also the computation time of the segmentation, 3D GLCM, and texture feature extraction was around 15 minutes for each case. However, given the reality of the ABC, texture feature based 3D GLCM is a more preferable approach to categorize ABC type than the recognized models.

## 5. Conclusion

In this paper, a new method based on multitexture features for abnormal cell classification of colorectal cancer is proposed. Real data of colorectal cancer was used to validate the discrimination between ABC features. ABC was segmented by active contour technique and then texture feature extracted from GLCMs. Significant texture features were selected based on ANOVA test. The best results were obtained when combining all features together and PCA was applied to get five PC features with accuracy of 92.59% to discriminate between ABC types. This result is promising to make a bride between image features and colorectal pathology which would lead to efficient medical diagnosis and treatment.

## Figures and Tables

**Table 1:** Average performance metrics (%) of three cancer cell types.

Metrics	BH	IN	Ca
JSC	76.12	75.92	81.56
DSC	86.44	86.31	88.21
FPR	07.61	05.03	06.32
FNR	18.08	20.26	16.11

**Table 2:** Grouping of texture features.

Groups	Offset	Phase	Features number
G_1_	1	13	156
G_2_	2	13	156
G_3_	**4**	13	156
G_4_	8	13	156
G_5_	1-2-4-8	13	624

**Table 3:** Comparison of ABC texture features (mean ± STD) between BH, IN, and Ca.

Feature	BH	IN	Ca	*p* value
*f*_1_	0.014 ± 0.009	0.007 ± 0.002	0.005 ± 0.001	<0.0001
*f*_2_	2.316 ± 0.202	2.382 ± 0.108	2.530 ± 0.059	<0.0001
*f*_3_	0.014 ± 0.004	0.016 ± 0.004	0.013 ± 0.001	<0.0001
*f*_4_	44.355 ± 21.172	33.183 ± 11.019	40.661 ± 11.612	<0.0001
*f*_5_	0.088 ± 0.048	0.079 ± 0.006	0.075 ± 0.005	<0.0001
*f*_6_	51.784 ± 8.455	49.623 ± 10.162	52.257 ± 5.684	0.0841
*f*_7_	17.032 ± 4.356	15.572 ± 0.764	17.075 ± 1.476	<0.0001
*f*_8_	44.355 ± 21.172	33.183 ± 11.019	40.661 ± 11.612	<0.0001
*f*_9_	−85.194 ± 589.163	375.391 ± 204.221	408.413 ± 229.755	<0.0001
*f*_10_	60430.702 ± 13323.791	54856.863 ± 19614.368	58506.092 ± 13952.284	0.0021
*f*_11_	0.064 ± 0.035	0.027 ± 0.008	0.026 ± 0.009	<0.0001
*f*_12_	0.215 ± 0.081	0.230 ± 0.038	0.201 ± 0.034	<0.0001

**Table 4:** Performance metrics (%) of ABC discrimination relied on texture feature selection.

Groups	Selected features (*p* < 0.01)	Accuracy	Sensitivity	Specificity
G_1_	35	66.66	55.55	77.77
G_2_	35	74.07	66.66	88.88
G_3_	35	77.77	88.88	94.44
G_4_	53	55.55	66.66	66.66
G_5_	158	74.07	77.77	94.44
PCs	5	92.59	100	94.44

**Table 5:** Accuracy classifier (%) of ABC using selected features.

Groups	BH	IN	Ca
G_1_	55.55	88.88	55.55
G_2_	66.66	88.88	66.66
G_3_	88.88	77.77	66.66
G_4_	66.66	44.44	55.55
G_5_	77.77	77.77	66.66
PCs	100	77.77	100

**Table 6:** Confusion matrix of the ABC.

Sample	DT			NN			NB		
	BH	IN	Ca	BH	IN	Ca	BH	IN	Ca
9 BH	9	0	0	8	1	0	9	0	0
9 IN	1	7	1	5	4	0	1	8	0
9 Ca	0	0	9	0	0	9	0	0	9

**Table 7:** *F*-score (%) of each ABC type.

Classifier	BH	IN	Ca
DT	94.73	87.50	94.73
NN	72.72	57.14	100
NB	94.73	94.11	100
